# Cyanobacterial Bloom and Toxin Identification in Austin, TX, USA Creeks

**DOI:** 10.17912/micropub.biology.001750

**Published:** 2026-02-26

**Authors:** Hanan Brower, Sahar Mahmood, Joana Ruiz-Escobar, Sara Bagheri, Omar Carrasco-Rubio, Luke Diggins, Anastasia Kuzmina, Wesley Tran, Kevin Zhu, Stuart Reichler

**Affiliations:** 1 Freshman Research Initiative, The University of Texas at Austin, Austin, Texas, United States

## Abstract

Cyanobacterial harmful algal blooms (cyanoHABs) can form in freshwater, and their toxins are harmful to flora and fauna, including humans. To assess the extent of cyanoHABs in urban waterways, seven creeks and Lady Bird Lake in Austin, TX USA were sampled from July to December 2024. Water chemistry was measured, cyanoHABs identified by microscopy, and cyanotoxins detected by LC-MS. Cyanobacteria, mostly genus *Oscillatoria* , was detected in all creeks sampled, and the primary cyanotoxin detected was cylindrospermopsin with levels varying between sampling locations and seasons. This study highlights the presence of cyanoHABs in creeks, and the potential risk they may pose.

**Figure 1. Presence of Harmful Cyanobacterial Blooms at Austin, TX USA Sampling Sites:**
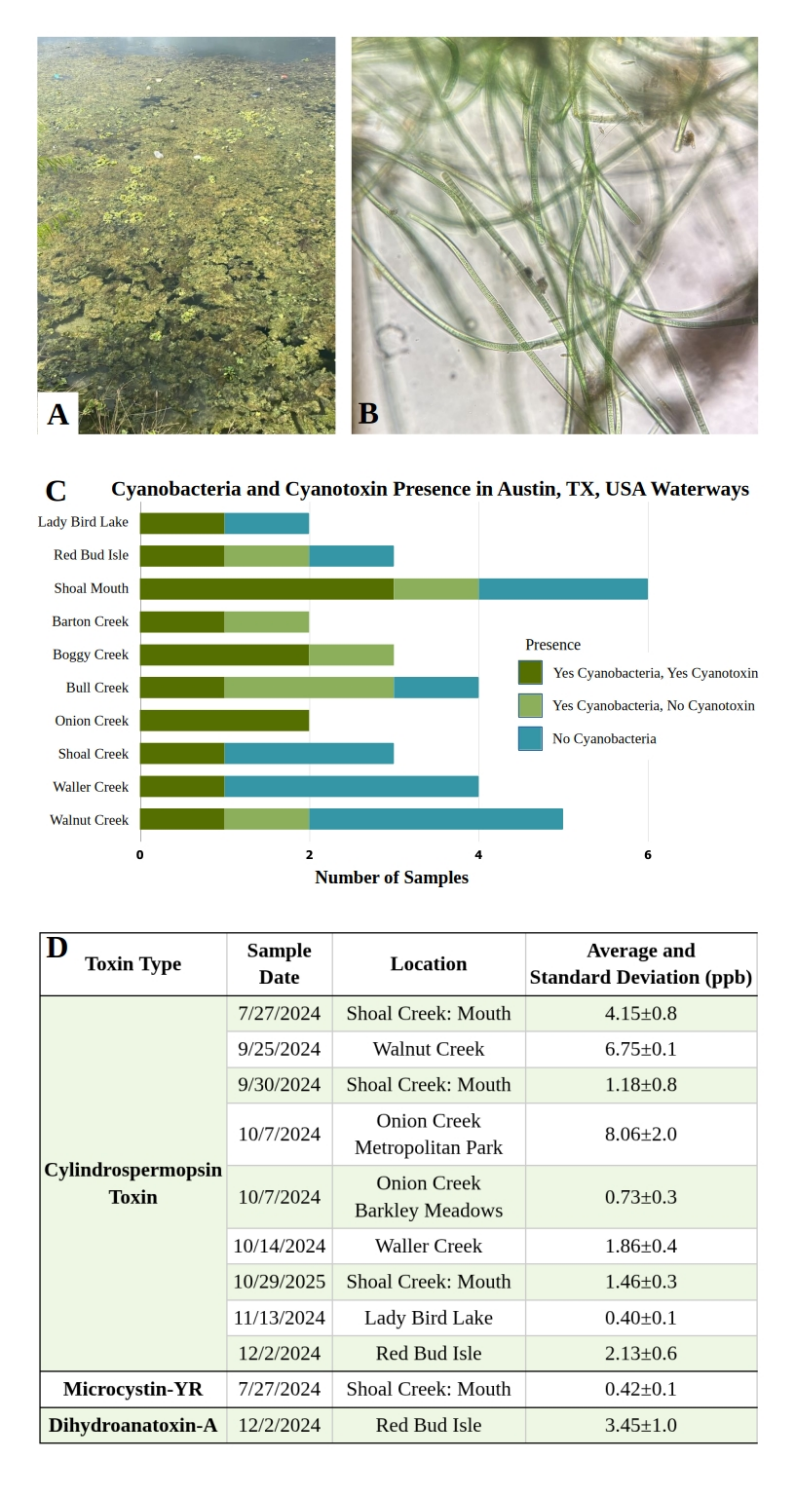
**A** . Extensive algal growth at Shoal Creek. This exemplifies the locations where algae samples were collected. **B** . Micrograph of *Oscillatoria* from a cyanoHAB collected at the mouth of Shoal Creek. Micrographs were photographed at 100X.&nbsp; **C.** Cyanobacteria presence and cyanotoxin detection by sample site. Cyanobacteria were detected by microscopy and cyanotoxins via LC-MS. Each site included in this study was sampled between two and six times. The bars represent all of the samples with blue indicating the number of samples where no cyanobacteria or cyanotoxin was detected; light green represents the samples that had cyanobacteria, but no cyanotoxin detected; and dark green are sites where both cyanobacteria and cyanotoxin was detected. **D** . Quantification of cyanotoxin type by sample location and date via LC-MS in ppb. All concentrations were determined using a standard of 5 ppb.

## Description

Cyanobacteria are a group of diverse aquatic photoautotrophic gram-negative bacteria that are becoming increasingly problematic due to their eutrophic growth capabilities and their production of cyanotoxins that can be harmful to flora and fauna (Carmichael, 1992; Ferrão-Filho et al., 2011; Gupta et al., 2013; Zepernick et al., 2023). Cyanotoxin production negatively impacts water use for human recreation, drinking, agriculture (Tanvir et al., 2021; Mutoti et al., 2023; Rocha et al., 2024), and negatively impacts other wildlife (Landsberg, 2002; Ash and Patterson, 2022; Dorantes-Aranda, 2023). The recent increase in cyanoHABs is a global phenomenon amplified by the effects of global warming and urbanization (Paerl and Barnard, 2020; Ranjbar et al., 2022).&nbsp;

The negative impacts of cyanoHABs have already been seen in Austin, Texas. In 2019, multiple dog deaths resulting from cyanotoxin poisonings were reported (Manning et al., 2020; City of Austin. https://www.austintexas.gov/page/algae-austins-waterways retrieved December 15, 2025). Because of this, the City of Austin (COA) has implemented increased surveillance of cyanoHABs and initiated public outreach efforts to raise awareness about the risks of cyanotoxin-related illness. The COA primarily monitors the Lady Bird Lake reservoir, part of the Colorado River (Perri et al., 2024; City of Austin. https://www.austintexas.gov/page/algae-austins-waterways retrieved December 15, 2025). Full coverage of Austin’s water bodies presents a challenge since monitoring is costly and resource-intensive (Gamez et al., 2019). In addition to these constraints, existing research has focused on cyanoHABs in ideal growth conditions such as large and still bodies of water (e.g. lakes and rivers) (Gugger et al., 2005; Huisman and Hulot 2005; Chen et al., 2017; Kozak et al., 2019; Lomeo et al., 2024; Perri et al., 2024). Despite reports of cyanoHABs in mountain streams, creek ecosystems are less frequently monitored (Huisman and Hulot, 2005; Gaysina et al., 2018; Genzoli and Kann, 2020).

Cyanobacteria were identified in all of the creeks sampled ( Figure 1C ). While cyanotoxins were detected in five of the seven creeks, the mouth of Shoal Creek, and sample sites in Lady Bird Lake ( Figure 1C ). Algal blooms were diverse and consisted of multiple cyanobacteria, filamentous green algae, diatoms, and other bacteria genera (Figure 2 B, C , and D). For the cyanobacteria, morphological characteristics were used to differentiate genera. *Oscillatoria* was found at all sites sampled. *Anabaena* was identified in only two samples.

Cyanotoxins were extracted from samples using solid-phase extraction and analyzed through triple quadrupole LC-MS. Cylindrospermopsin was the only cyanotoxin detected in Austin creeks ( Figure 1D ). Dihydroanatoxin-a (dhATX) was only present at the Red Bud Isle (lake) site ( Figure 1D ). Because these cyanotoxins vary in toxicity, understanding the distribution of the different cyanotoxins across locations is important for preventing future cyanotoxin-caused illnesses. Cylindrospermopsin is known to cause hepatic illness in mammals (Buratti et al., 2017). Cylindrospermopsin poisoning was attributed to cattle deaths observed in Queensland, Australia in 1992 (Thomas et al., 1998). In humans this hepatic illness is shown to be reversible with medical treatment (Buratti et al., 2017). The Environmental Protection Agency (EPA) recommends cylindrospermopsin concentrations do not exceed 15 ppb in recreational water (Ross, 2019). dhATX is a neurotoxin directly linked to fatal poisonings in Austin, TX dogs (Fredrickson et al., 2023). Neither the World Health Organization (WHO) nor the EPA provide recommendations for dhATX in recreational water, however, dhATX, a congener of anatoxin (ATX), was found to be up to four times more lethal in mice than ATX when ingested orally (Puddick et al., 2021; Fredrickson et al., 2023).

With additional ongoing sampling, we are working on elucidating conditions that lead to the production of the different cyanotoxins. The concentrations of detected cyanotoxins were mostly less than the 5 ppb standard ( Figure 1D ). Barton and Boggy Creek did not have detectable levels of cyanotoxin, despite cyanobacteria being seen in both site’s mat samples. CyanoHABs are present in Austin creeks and produce detectable concentrations of cylindrospermopsin cyanotoxin.

Since these creeks are commonly used for recreational activities, the presence of cyanoHABs poses a potential danger to the public. Monitoring creeks during peak bloom seasons can help limit the danger by allowing warnings to be posted when cyanotoxins are detected. Since cyanoHABs monitoring is resource intensive, further elucidation of the connection between environmental factors and cyanoHABs can allow predictive modeling using a more easily quantified variable, like water chemistry (Figure 2F), allowing municipalities to warn people when cyanoHABs are likely to be present.

## Methods

**Site Characterization**

Sites were chosen based on their recreational activity use and access for sampling. The sites Shoal Creek Mouth, Lady Bird Lake, and Red Bud Isle were selected because of previous reports of dog illnesses and deaths at these locations (Manning et al., 2020). Creek sites were chosen as locations where a clear downstream flow could be seen and the site was clearly above the water level at the mouth. Mouth sites were determined to be at the mouth of the creek and contained a mixture of lake and creek water.&nbsp;

**Sample Collection and Water Chemistry Measurements**

Sites were sampled for algal and possible cyanoHABs between June 28, 2024, and December 2, 2024 (Figure 2E). Sites are Austin, TX, USA creeks and reservoir locations that are accessible to the public for recreational use. Upon arriving at the site, floating and shiny algal mats were sought. Mats were collected by hand or by scraping the creek bed and placed in 500 mL acid-washed plastic amber bottles along with creek water. Water temperature (℃), dissolved oxygen (ppm), pH, and conductivity (μS/cm) were collected on-site using a YSI Multiparameter Probe. Water chemistry analysis was performed within one hour using a Chemetrics V-2000 multi-analyte photometer to determine nitrate (NO _3_^–^ ) and phosphate (ortho, PO _4_^3–^ ) concentrations. Samples were stored at room temperature for one hour until imaging and extraction were performed.

**Microscopy**

Köhler illumination for microscope imaging was achieved using a ZEISS Axiolab 5 Microscope with 50X or 100X magnification.

**LC-MS Analysis**

The extraction and detection of cyanotoxins followed the protocol from Fredrickson et al., 2023. Samples were processed in reduced light to minimize photolysis. Using a vacuum manifold, Waters C-18 Plus SPE cartridges were conditioned with methanol (100% Optima MeOH) and ultra-pure water. Water samples from the bottles containing collected algae were introduced and passed through the cartridge. Methanol was used to elute the samples, which were evaporated using nitrogen gas and resuspended in 5% Optima MeOH, before analysis on a Shimadzu 8060 triple quadrupole LC-MS (Figure 2 G-J). The cyanotoxins tested for are: microcystin-LR (MC-LR), anatoxin-a (ATXa), cylindrospermopsin (CYR), saxitoxin (STX), dihydroanatoxin-a (dhATX), homoanatoxin-a (HTX), microcystin-YR (MC-YR), microcystin-RR (MC-RR), microcystin-LA (MC-LA), and nodularin (NOD). These 10 cyanotoxin standards were added at 5 ppb, and concentrations were calculated through a standard curve using 0 and 5 ppb by the Shimadzu Lab Solutions™ software.

## Reagents

Nitrate Vacu-vials Kit (K-6913)

Reagents: Hydrochloric Acid (S-6901) and Zinc foil packet

Phosphate Vacu-vials Kit (K-8513)

Reagents: Glycerol and Stannous Chloride Dihydrate (S-8500)

## Data Availability

Description: Figure 2 A: Floating mat collected from Onion Creek. B, C, and D: Micrographs of algal blooms collected in Austin creeks. Micrographs were photographed at 100X. These micrographs were taken from samples collected at Bull Creek (B, C), and Red Bud Isle (D). E: Sample sites for algal mat and water collection. Map created with Google Earth mapping software. F: Graphical summary of water quality variables. Median and interquartile range (IQR) was taken for the environmental factors measured for each sample site during the period of July to November 2024. Variables listed are nitrates (NO3 in ppm), phosphates (PO4 in ppm), temperature (℃), dissolved oxygen (DO in ppm), and conductivity (μS/cm). G: LC-MS data showing positive identification of cylindrospermopsin cyanotoxin at Onion Creek 10/11/2024. H: The relevant signal from a multi-reference cyanobacteria standard matches well to our sample peak (G). The x-axis shows retention time and the y-axis shows relative signal intensity. I: LC-MS data showing positive identification of dhATX in Red Bud Isle 12/2/2024. J: The relevant signal from a multi-reference cyanobacteria standard matches well with our sample peak (I). The x-axis shows retention time and the y-axis shows relative signal intensity.. Resource Type: Image. DOI: https://doi.org/10.22002/09ea9-n1173
